# Supplementary material for: Mitochondrial D-loop sequence variation and maternal lineage in the endangered Cleveland Bay horse
Source: PLoS One. 2020 Dec 3;15(12):e0243247. doi: 10.1371/journal.pone.0243247 (PMC7714183; doi:10.1371/journal.pone.0243247)
Supplement: Attachment — Submitted filename: R1ResponseToReviewersmtDNACBH.docx [file pone.0243247.s001.docx]

Reviewer #1: This paper presents analysis of mtDNA typing of the Cleveland Bay horse, one of the world's most endangered horse breeds. The paper is well written and appropriate methods are used. I have only a couple of comments. One, the mtDNA haplotype nomenclature of Jansen has largely been replaced by that of Achilli et al. 2012. Using Jansen is ok but Achilli should also be used concurrently.

The nomenclature from Achilli has been incorporated within **Table 2.**

The diversity of mtDNA in the CB is consistent with the low nuclear DNA variation that has recently been reported and the high inbreeding level within the breed. It is interesting how well the maternal lines predicted by pedigree analysis are aligned with the mtDNA haplotypes. I also am somewhat surprised by how high the proportion of haplotypes representing horses from the British Isles is. Within breed ancestry representations are often quite diverse representing past intermixing of horses prior to breed formation. I also will say that the diversity shown does not indicate that the Chapman horse is not the direct link to all parts of the CB ancestry as there is no knowledge of the make-up of that horse.

My one negative comment is not actually a criticism of the paper. This paper has a great deal of very specific information about the make-up of the individuals that comprise the breed. This has almost no meaning to most potential readers but is valuable information for the specialist. The great detail of the specific maternal lines and groups within the breed make certain sections of this paper very difficult to follow. In fact the paper presents little that is new except in reference to the breed. The paper might be more appropriate for a different journal although I don't have a specific recommendation for one.

Reviewer #2: The current study aimed to establish maternal lineages within the Cleveland Bay Horse breed and detect relationships with other domestic horse breeds. The Cleveland Bay Horse is a very important breed and known to have low genetic diversity. The authors used typical methods for the data analysis of part of the D-loop. I think sequencing the full D-loop would be more informative especially with the high level of similarity seen among tested samples. Although the results and discussion were well written, the approach and results are lacking novelty, very specific to horses and not applicable for novel discoveries in other species. The overall outcomes of the current study might be interesting and useful for a narrow group of readers. More importantly, I wish the authors have included the finding of this study with their previous paper. The story could be then much informative by integrating the autosomal genetics findings in the light of the mtDNA sequencing instead of having them separated in two manuscripts.

We thank the reviewers for their comments, and their careful assessment of the manuscript.
